# Human embryonic stem cell-derived retinal pigment epithelium transplants as a potential treatment for wet age-related macular degeneration

**DOI:** 10.1038/s41421-018-0053-y

**Published:** 2018-09-11

**Authors:** Yong Liu, Hai Wei Xu, Lei Wang, Shi Ying Li, Cong Jian Zhao, Jie Hao, Qi You Li, Tong Tao Zhao, Wei Wu, Yi Wang, Qi Zhou, Cheng Qian, Liu Wang, Zheng Qin Yin

**Affiliations:** 1 Southwest Hospital/Southwest Eye Hospital, Third Military Medical University (Amy Medical University), Chongqing, 400038 China; 2 State Key Laboratory of Stem Cell and Reproductive Biology, Institute of Zoology, Chinese Academy of Sciences, Beijing, 100101 China; 3 University of Chinese Academy of Sciences, Beijing, 100049 China; 4 Institute of Pathology and Southwest Cancer Center, Southwest Hospital, Third Military Medical University(Amy Medical University), Chongqing, 400038 China

## Abstract

Stem cell therapy may provide a safe and promising treatment for retinal diseases. Wet age-related macular degeneration (wet-AMD) is a leading cause of blindness in China. We developed a clinical-grade human embryonic stem cell (hESC) line, Q-CTS-hESC-2, under xeno-free conditions that differentiated into retinal pigment epithelial cells (Q-CTS-hESC-2-RPE). A clinical trial with three wet-AMD patients was initiated in order to study the safety and tolerance to Q-CTS-hESC-2-RPE cell transplants. The choroidal neovascularization membrane was removed and then a suspension of 1 × 10^6^ Q-CTS-hESC-2-RPE cells were injected into a subfoveal pocket. The patients were followed for 12 months during which no adverse effects resulting from the transplant were observed. Anatomical evidence suggested the existence of new RPE-like cell layer in the previously damaged area. Visual and physiological testing indicated limited functional improvement, albeit to different degrees between patients. This study provides some promising early results concerning the use of transplanted hESC-RPE cells to alleviate wet-AMD.

## Introduction

Age-related macular degeneration (AMD) is the leading cause of blindness among the elderly in many countries^1^. AMD can be classified as a neovascular disruption (wet-AMD) or atrophic damage to the retinal pigment epithelium (RPE) (i.e. dry-AMD)^2^. RPE cells are crucial to the regulation of homeostasis in the micro-environment between choroid and photoreceptors^3^. Therefore, the occurrence of degeneration and pathological changes in the RPE at the center of retina is closely related to AMD^2^.

The prevalence rates of early (mainly refers to drusen formation in the subretinal space) and late (choroidal neovascularization (CNV) or geographic atrophy occured) AMD in Chinese individuals 50 years of age or older were estimated to be 9.5% and 1.0%, respectively. Among these, wet-AMD predominated, and its medical care has become a major challenge^4,5^. Current treatments, such as the intraocular injection of anti-vascular endothelial growth factor drugs (VEGF), have revolutionized the clinical management of wet-AMD; however, monthly injections are tedious for patients and only control neovascular lesions. CNV in advanced wet-AMD causes substantial damage to the RPE and photoreceptors^6^. Many patients may still lose their vision because of late diagnosis or inadequate treatment^7^. Although subretinal surgery to remove CNV has been attempted in wet-AMD, it did not result in improved visual outcomes^8–10^, and this suggested that photoreceptor function was not restored after CNV removal due to a lack of support from healthy RPE cells.

While a significant number of RPE cells and photoreceptors are lost/impaired in AMD, the remaining cells, including some photoreceptors, bipolar cells, and ganglion cells, are thought to maintain viable retinal connections, albeit these connections may have been substantially modified^11,12^. Thus it seems appropriate to pursue a strategy that repairs or replaces the damaged RPE and photoreceptor layers^13^. Replacing degenerative or dead RPE cells with healthy RPE cell transplants in animal models of retinal degeneration indicated that dying photoreceptors could be rescued with an associated improvement in vision^14,15^. Allogeneic RPE sheets derived from human fetuses and autologous peripheral RPE cells have been used as transplant material in AMD patients over the past 20 years^16,17^. However, these clinical trials have been hindered due to the limited cell sources for transplants and the higher risk associated with the complicated surgical procedures needed to obtain the cell. Studies searching for more abundant and possibly robust RPE cell sources that overcome these limitations are a promising line of research.

Several types of stem cells, such as adult stem cells, embryonic stem cells (ESCs), and induced pluripotent stem cells (iPSCs), have been successfully differentiated into RPE cells in vitro^18^. Recently, RPE cells derived from human embryonic stem cells (hESCs) have been used clinically to treat dry-AMD and Stargardt’s disease^19–21^. Although the long-term efficacy remains to be determined, it has been shown to be a safe treatment for AMD^22^. The procedures of stem cell-based cell therapy for treatment of wet-AMD are more complicated than those for dry-AMD. Because the CNV membrane must be removed before cell transplantation in wet-AMD, the procedure is associated with a higher risk of massive hemorrhage and retinal detachment. Mandai et al^22^ reported the results of iPSC-derived RPE sheet transplantation in one patient with wet-AMD. After a 1-year follow-up, the patient’s vision had neither improved nor worsened. Recently, Cruz et al. reported that transplantation of hESC-RPE patch in the retinas of two severe wet-AMD patients and proved the safety of hESC-RPE patch in the treatment for AMD patients^23^. In the present study, using the established clinical-grade hESC line (Q-CTS-hESC-2) according to Chinese regulations and hESC-derived RPE (hESC-RPE) cells^24,25^, we delivered the suspension of these clinical-grade hESC-RPE cells in the subretinal spaces of three wet-AMD patients to test the safety and feasibility as a therapeutic strategy for wet-AMD.

## Results

### Human ESC-derived RPE Cells transplanted into SCID mice and RCS rats

It took ~125 days and three passages to efficiently induce hESCs to differentiate into RPE cells that were suitable for clinical use (Supplementary Fig. S1a, b). Immunofluorescent staining for RPE markers showed that >99% cells were positive for MITF, ZO-1, Bestrophin-1, REP-65, and CRALBP (Supplementary Fig. S1c), and most cells expressed PAX6 (96.6% ± 2.4%, *n* = 3). Fluorescent-activated cell sorting (FACS) analysis indicated that the purity of the hESC-RPE cell cultures was >99%, as indicated by the number of cells labeled positive for RPE markers such as Bestrophin-1 (99.2% ± 0.2%, n = 3), RPE-65 (99.3% ± 0.2%, *n* = 3) and CRALBP (99.2% ± 0.5%, *n* = 3) (Supplementary Fig. S1e). In addition, the expression level for pluripotency genes (OCT4, Nanog, and Sox2) was markedly decreased by 10–100 fold compared to ESCs; a marker of neuroectoderm differentiation (PAX6) and RPE markers (MITF, Bestrophin-1, REP-65 and CRALBP) were expressed at higher levels compared to ESCs (100–1000 times, *n* = 3, Supplementary Fig. S1d). We used phagocytosis of photoreceptor outer segment (POS) and labeling of Na/K-ATPase to test in vitro function of hESC-RPE cells. As expected, RPE cells could phagocytize POS and Na/K-ATPase was expressed at the apical membrane in RPE cells (Supplementary Fig. S2). The RPE lot we used had a melanin content of 4.75 ± 0.70 pg per cell (*n* = 3), which was similar to that in the work from Schwartz et al.^19,20^. Whole exome sequencing of Q-CTS-hESC-2-RPE was performed, four related mutation sites were identified based on the results of the sequencing. These four mutation sites were verified with Sanger PCR method, revealing no mutations in these candidate sites for oncogenes or tumor suppressor genes in these Q-CTS-hESC-2-RPE cells (Supplementary Fig. S3).

Royal College of Surgeons (RCS) rats, a well-known model of retinal degeneration due to a phagocytic defect in the RPE cells, received a subretinal injection of Q-CTS-hESC-2-RPE cells (1 × 10^5^ cells in 2 μl PBS) according to the method previously described by our lab^25^ (Supplementary Fig. S4). Functional electroretinogram (ERG) tests showed that the amplitude of the b-wave was significantly higher in the treated RCS rats compared to the normal saline-treated group 4 and 8 weeks after cell transplants (295.3 ± 68.1 μV vs. 50.2 ± 1.1 μV, *p* < 0.001, 4 weeks post-transplant; 117.7 ± 3.8 μV vs. 16.8 ± 5.5 μV, *p* < 0.001, 8 weeks post-transplant). In addition, eyes with transplants maintained a significantly thicker outer nuclear layer (ONL) compared to the control group (57.3 ± 4.1 μm vs. 26.2 ± 1.1, *p* < 0.01, 4 weeks post-transplant; 28.29 ± 1.32 μm vs. 19.54 ± 1.21 μm *p* < 0.05, 8 weeks post-transplant). Biosafety analysis confirmed the pathogen- and virus-free status of the Q-CTS-hESC-2-RPE cells, based on fungal, bacterial, and viral examination, and endotoxin detection following the China Food and Drug Administration (CFDA) guidelines (Supplementary Table S1). Tumors were not present in immunodeficient (SCID) mice subcutaneously injected with Q-CTS-hESC-2-RPE cells at 3 months post-transplant (*n* = 46). In contrast, undifferentiated Q-CTS-hESC-2 cells did develop into teratomas in 65% of the animals (*n* = 13/20; Supplementary Fig. S5). Taken together, our results suggested our hESC-RPE cells could be valuable for the foundation of an international standard for clinical-grade cells for therapy.

### Clinical trial of hESC-RPE transplantation in wet-AMD patients

**Fig. 1:**
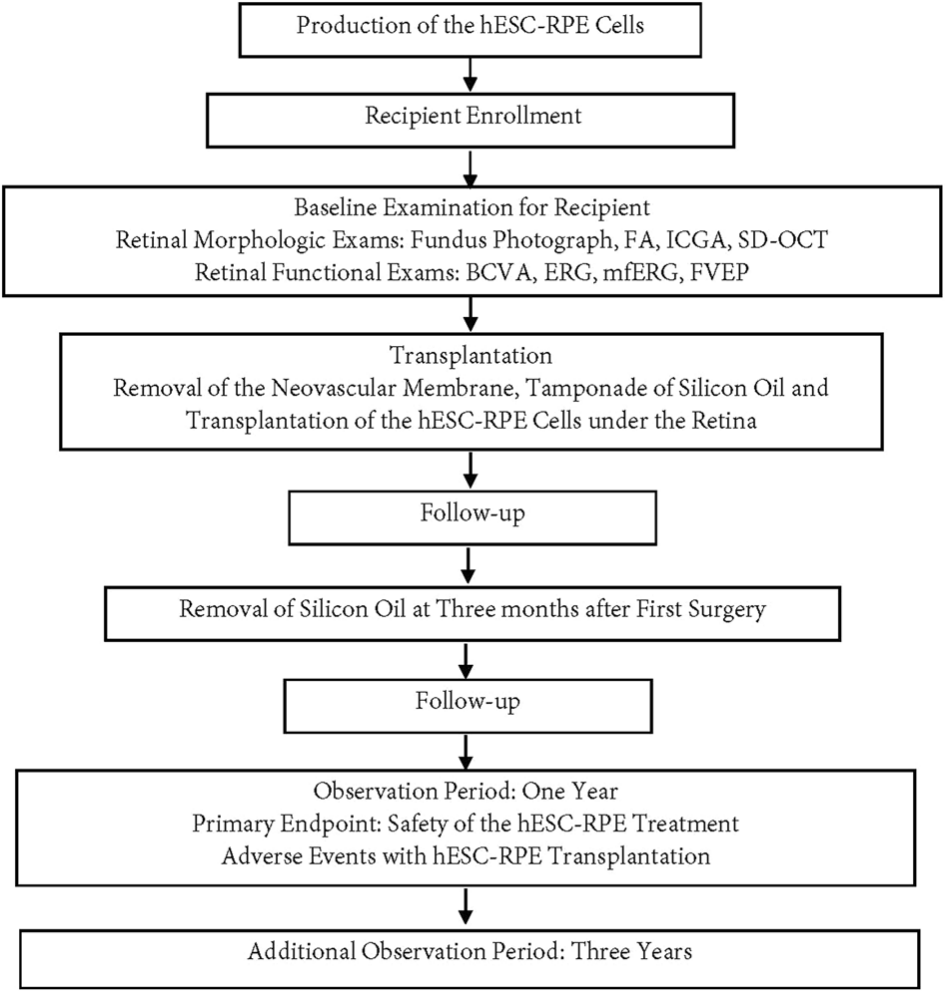
Flow chart showing the experimental protocol

We designed a trial profile to assess the suitability of wet-AMD patients before enrollment in the Q-CTS-hESC-2-RPE cell transplant trail (Fig. 1 and Supplementary Table S2). After receiving approval by the Ethics Committee of the Southwest Hospital (No. 2015-18) and registration on the website http://clinicaltrials.gov (NCT02749734), we carried out a phase I clinical study focusing on the safety of the Q-CTS-hESC-2-RPE cell transplants into the subretinal space for the treatment of wet-AMD.

#### Patient #1

**Fig. 2:**
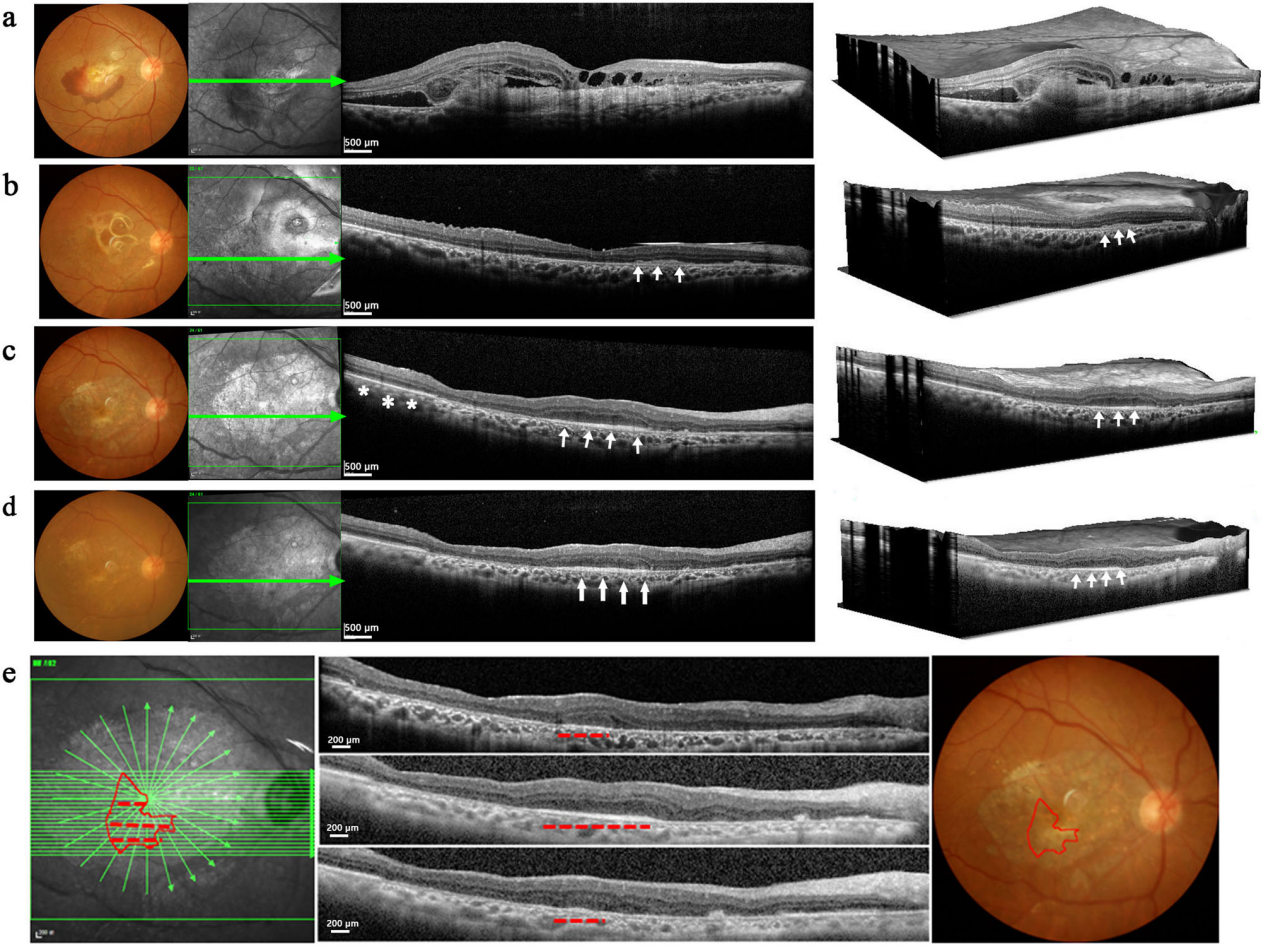
Morphological changes in patient #1 following CNV removal and hESC-RPE cell transplantation. **a**–**d** show pre- and postoperative color fundus photographs (column 1); en-face structural OCT (column 2) in which the green arrows correspond to the highly-sampled OCT b-scans vertical sectional views in column 3; column 4 shows 3D reconstruction images of the same retinal region. **a** A large fibrotic CNV and hemorrhage were observed in the subfoveal space preoperatively. **b** 1 month after surgery the fundus images show a complete removal of the CNV, and an almost intact Bruch’s membrane. Note the cell clusters (indicated by white arrows) evident as a dense medium reflective mass in the region of baseline RPE atrophy. **c** At 6 months, macular edema was not observed in the region of the fovea. A highly reflective RPE-like cell layer was observed in the transplant region (white arrows). This layer was similar to the healthy RPE outside the region of the transplant (white asterisks). **d** At 12 months the highly reflective RPE-like cell layer was still present in the transplant area (white arrows). The photoreceptor layer above this region shows better anatomical preservation compared to adjacent regions. Panel e shows the mapping strategy using OCT scanning to determine the distribution of grafted cells; 61 horizontal lines were evenly scanned while focusing on the transplantation area. An RPE-like cell layer extended from the 20th to the 38th scan (red dashed lines in the OCT images). SD-OCT spectral domain ocular coherence tomography, CNV choroidal neovascularization, RPE retinal pigment epithelial

Patient #1 was a 60-year-old male with advanced wet-AMD and an initial best-corrected visual acuity (BCVA) of hand movements (HMs) for the right eye (study eye) and a BCVA for the left eye of 20/30 using a Snellen visual chart. Previously, the patient had received a total of five intravitreal injections of an anti-VEGF drug in the right eye. However, visual acuity gradually decreased prior to his initial visit to our hospital. An eye examination showed a fibrotic CNV membrane beneath the fovea (Fig. 2a).

The CNV membrane was completely removed without bleeding, and then a suspension of Q-CTS-hESC-2-RPE cells (1 × 10^6^ cells in 100 μl normal saline) were successfully delivered into the submacular area (Supplementary Movie S1) without cell leakage. An immunosuppression regimen was rigorously implemented for 4 months and did not give rise to any serious adverse events. There were no signs of aqueous flare or vitreal cells beyond what would be typically observed in the 2-week postoperative period, and this suggests that subsequent cell leakage from the transplant area was minimal. Ocular fundus photographs showed no hemorrhaging in the fovea 1 month after surgery. The spectral domain ocular coherence tomography (SD-OCT) indicated that the subretinal membrane had been completely removed, and the central macula depression was present without edema. Injected cell clusters with a dense medium reflective mass were observed in the area previously showing RPE damage (Fig. 2b). By 6 months, a highly reflective RPE-like cell layer was readily visible in the same area and appeared to be similar to the healthy RPE layer (Fig. 2c). In addition, the overlying neuroretinal lamination supported by the new RPE-like layer was 279 μm thick, and maintained its cellular integrity; the adjacent neuroretinal tissue without RPE cell support was only ~207 μm thick and showed ONL thinning. The horizontal SD-OCT scanning images were reconstructed to map the distribution of grafted cells using Image J (Fig. 2e). The transplant area had a roughly pie-shaped distribution when reconstructed from 19 OCT slices. At 12 months, the transplant area containing RPE-like cells remained stable (Fig. 2d). Our clinical tracing method indicated that the transplanted cells existed for at least 12 months in this patient.

**Fig. 3:**
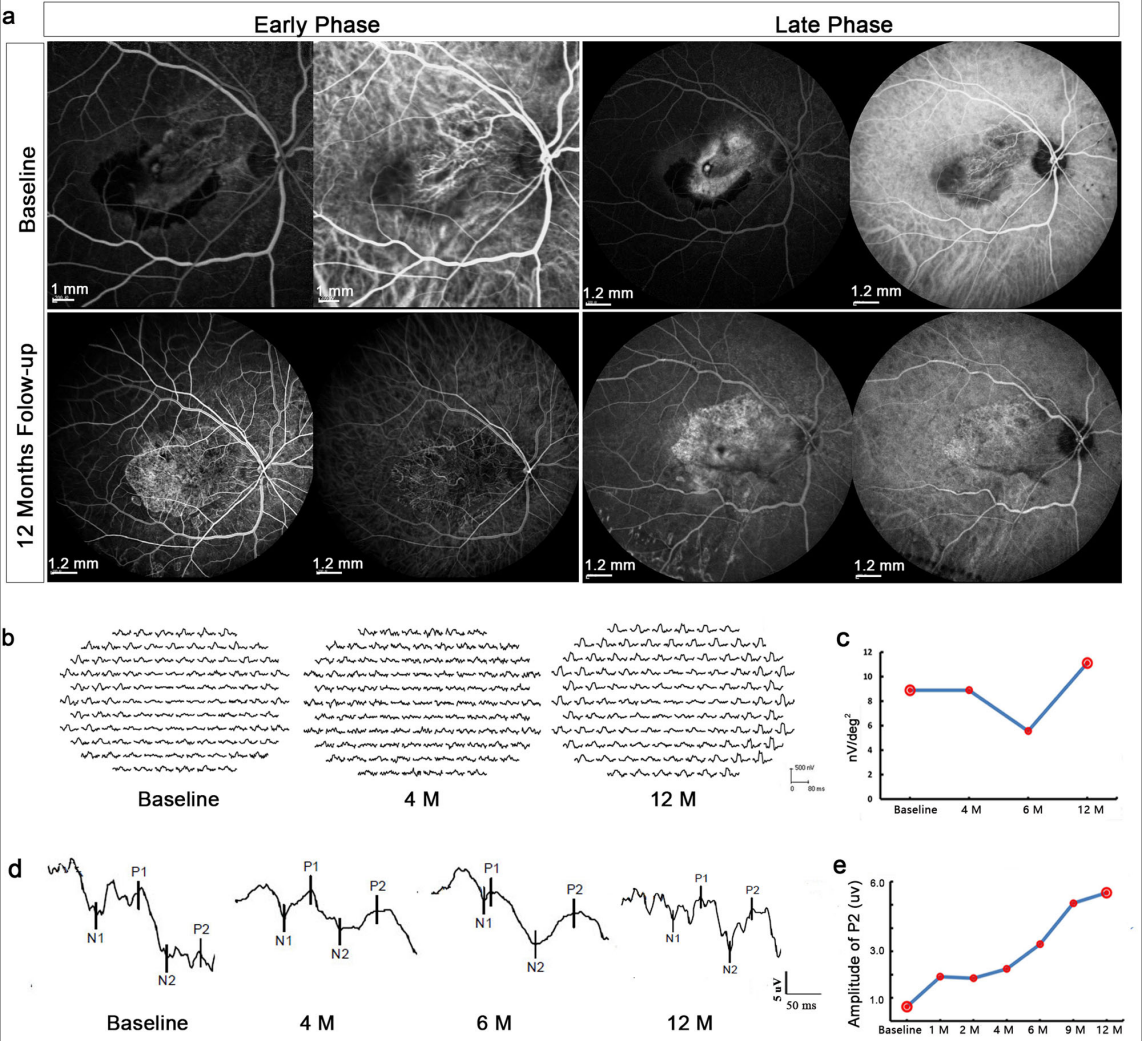
Angiographic characteristics and physiological changes in Patient #1. **a** Retinal and choroidal vascular changes as seen with fundus fluorescein FA (left side of pair) or ICGA (right side of pair). The preoperative test showed the subfoveal choroidal neovessels beneath the CNV membrane (early phase) and leakage with hyperfluorescence that obscured the boundaries of the lesion (late phase). One year later, new vessel growth was not observed in the ICGA, nor any additional leakage observed in the FA. **b** The mfERG waves before (baseline) and after (4 and 12 months follow-up) transplantation. **c** Analysis of amplitude changes for the central hexagon (ring 1) that mainly represents foveal visual function. The standard measurement for mfERG amplitude is the trough-to-peak amplitude (nv) measured between N1 and P and given as the amplitude density nv over its hexagon in degrees (nv/deg^2^). **d** The waveform of FVEP at different time points after transplantation. **e** Graph shows P2 amplitude changes induced by FVEP. FA fluorescein angiography, ICGA indocyanine green fluorescein angiography, mfERG multifocal electroretinography, FVEP flash visual evoked potentials

We used fundus fluorescein angiography (FA) and indocyanine green fluorescein angiography (ICGA) respectively, to determine whether recurrent CNV or additional vascular leakage had occurred in the retinal and choroidal vessels after the surgery (Fig. 3a). The preoperative test showed the subfoveal choroidal neovessels beneath the CNV membrane layer (early phase) and leakage with hyperfluorescence (late phase) that obscured the boundaries of the lesion. In the 12-month follow up period, we did not observe any new exudative neovascularization, vessel leakage, or sustained local retinal inflammation in the diseased area.

Visual acuity changes were assessed by means of BCVA, multifocal electroretinography (mfERG), and flash visual evoked potentials (FVEP). One month after the transplant, the patient’s BCVA increased from HMs to 20/125, and he could read 41 letter in the Early Treatment Diabetic Retinopathy Study (ETDR) visual acuity test (Table 1). A second surgical operation was performed to remove silicon oil 3 months after transplantation. However, a cataract gradually developed necessitating cataract extraction and intraocular lens implantation at 9 months post-transplant. The BCVA showed a further increase to 20/400 (13 ETDR letters; Table 1) at 12 months post-transplant.

**Table 1:** Changes in visual acuity after hESC-RPE transplantation in patients with wet AMD

	Patient 1		Patient 2		Patient 3	
	Operated Eye	Fellow Eye	Operated Eye	Fellow Eye	Operated Eye	Fellow Eye
Baseline	0^a^ (HM^b^)	74 (20/25)	5 (HM/20cm)	77 (20/25)	10 (20/400)	68 (20/40)
1 Month	41 (20/125)	76 (20/25)	12 (20/1000)	80 (20/25)	24 (20/400)	72 (20/32)
2 Months	52 (20/150)	85 (20/20)	12 (20/1000)	82 (20/25)	23 (20/400)	72 (20/40)
3 Months Silicon Oil Removal	38 (20/400)	83 (20/25)	7 (20/1000)	80 (20/25)	23 (20/400)	76 (20/32)
6 Months	15 (20/400)	85 (20/25)	8 (20/400)	79 (20/25)	19 (20/400)	78 (20/32)
9 Months Cataract Extraction	12 (20/400)	86 (20/25)	8 (20/400)	79 (20/25)	29 (20/400)	78 (20/32)
12 Months	13 (20/400)	83 (20/25)	16 (20/400)	80 (20/25)	36 (20/400)	78 (20/32)

^a^Represents the visual acuity tested using the Early treatment diabetic retinopathy (ETDR) chart

^b^Represents the visual actuty tested using a Snellen’s chart, HM -hand movement

Because Q-CTS-hESC-2-RPE cells were transplanted in the region of the macula, we used the response density of ring 1 of the mfERG to analyze macular function^26^. The response density was 8.9 nv/deg^2^ preoperatively, and remained stable up to 4 months post-transplant but had decreased to 5.6 nv/deg^2^ by 6 months, probably due to the presence of the cataract given the marked improvement to 11.1 nv/deg^2^ seen at 12 months (Fig. 3b, c). The most consistent and robust component of the FVEP in normal adults is the P2 peak^27^ and P2 amplitude can be used to infer conductive function along the length of the visual pathway. P2 amplitude improved over the 12 months study period (0.5 μv preoperatively vs. 6.0 μv 12 months post-transplant, Fig. 3d, e).

#### Patient #2

**Fig. 4:**
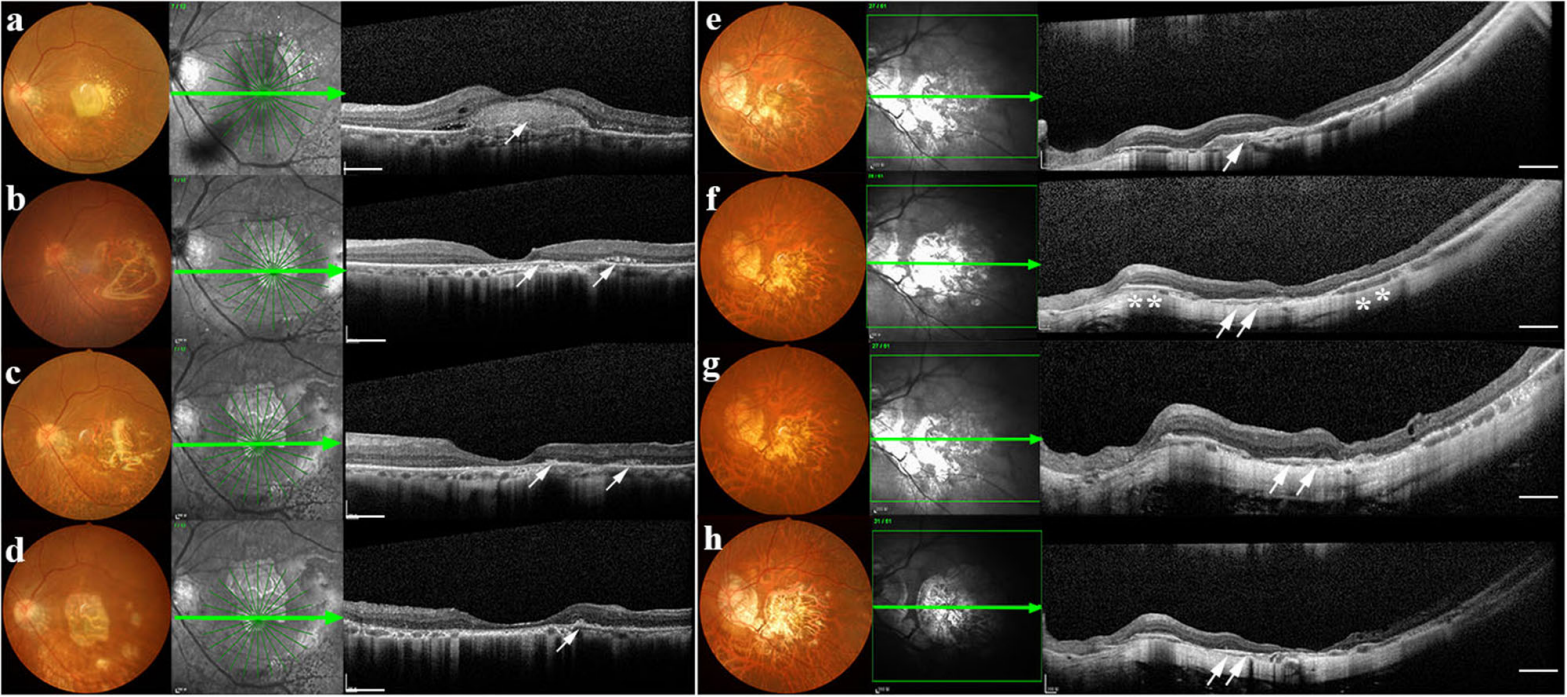
Morphological changes after CNV removal and hESC-RPE transplantation in Patients #2 and #3. **a**–**d** Patient #2: pre- and postoperative color fundus photographs (left panel), en-face structural SD-OCT images (middle panel) in which the green arrow corresponds to the highly sampled SD-OCT b-scan in the right-most panel. **a** The subretinal neovascular membrane could be clearly seen prior to the transplant (white arrow). **b** Four months after surgery, the subretinal neovascular membrane was gone and there is no indication of macular edema. Cell clusters (white arrows) could be observed within and adjacent to macular area. **c** Six months post-transplant, cell clusters (white arrows) were seen in the graft region. **d** By the 12 month follow-up, the transplanted cell clusters appeared to have merged (white arrows). **e**–**h** Patient #3: (**e**) A subretinal neovascular membrane was seen before surgery (white arrow). **f** 4 months after surgery; no hemorrhage or edema was observed in the fovea. An RPE-like cell layer had formed under the macular zone (white arrows) that was similar to the host RPE layer (white asterisks). **g** 6 months later the highly reflective RPE-like cell layer was still present in the graft area. **h** 12 months later there still appeared to be evidence for an RPE-like cell layer in the graft area (white arrows). Scale bar = 1 mm. SD-OCT spectral domain ocular coherence tomography, RPE retinal pigment epithelial

Patient # 2 was a 71-year-old female with an initial BCVA of HMs in the left eye (study eye) and 20/25 in the right eye. Initial observations indicated subfoveal scar tissue formation with obsolete hemorrhage (Fi. 4a), thus this patient was advised not to proceed with anti-VEGF therapy. We successfully performed CNV removal and a Q-CTS-hESC-2-RPE cell subretinal injection. Retinal photographs did not show any signs of hemorrhage in the fovea 4 months post-transplant, and SD-OCT scanning indicated the subretinal neovascular membrane had been completely removed. Some small clusters of injected cells were identified adjacent to the central fovea (Fig. 4b) and these cellular masses were still clearly present in the graft region at 6 months (Fig. 4c). These cell clusters tended to merge with the supporting Bruch’s membrane (Fig. 4d) by 12 months. SD-OCT examination at the 12 month follow-up visit showed that central fovea had maintained its morphologic depression without edema. FA and ICGA at this time also showed no additional fluorescent leakage from retinal or choroidal vessels (Supplementary Fig. S6), indicating a lack of obvious retinal inflammation or recurrent CNV.

BCVA increased slightly from HMs to 20/1000 (12 ETDR letters) 1 month after post-transplant. This patient also had silicon oil removal and cataract extraction, respectively, 3 and 9 months post-transplant. Similar to patient #1, visual acuity showed a further improvement to 20/400 (16 ETDR letters) following cataract removal. The central response density of the mfERG was 5.6 nv/deg^2^ preoperatively, then increased to 10.0 nv/deg^2^ at 6 months, but had decreased to 7.8 nv/deg^2^ by 12 months post-transplant. The P2 amplitude of the FVEP was 10.8 μv at baseline and had increased to 21.2 μv by 6 months, but had declined to 14.2 μv by 12 months.

#### Patient #3

Patient #3 was a 60-year-old female with an initial BCVA of 20/400 with 10 ETDR letters for the left eye (study eye) and 20/40 for the right eye. She received two intravitreal injections of anti-VEGF prior to enrollment. However, the subretinal neovascular membrane still remained (Fig. 4e). Four months after the Q-CTS-hESC-2-RPE cell transplant (Fig. 4f) a new RPE-like layer with a higher reflection had formed under macular zone, and this layer was still present at the 6 month follow-up examination (Fig. 4g). Similar to patient #1, retinal regions outside the area of the initial hESC-RPE cell transplant showed signs of better preservation compared to areas without an RPE layer, which showed atrophy and a loss of integrity within the ONL and outer retinal layers. The RPE-like layer was still observed at 12 months (Fig. 4h); however, the retina appeared to have undergone additional atrophy when compared to that seen at 6 months. Although the BCVA assessed by ETDR had increased from 10 to 24 letters after 1 month post-transplant, with further improvement to 36 ETDR letters by 12 months following silicon oil removal and cataract extraction (at three and nine months respectively), the Snellen visual acuity remained at 20/400 over the 12-month period (Table 1). The mfERG ring 1 amplitude density was too low to reliably record preoperatively and remained close to baseline levels for 6 months, but had increased to 11.1 nv/deg^2^ by 12 months (Supplementary Fig. S7). The P2 amplitude of the FVEP was 9.9 μv at baseline and had increased to17.4 μv by 6 months, but by 12 months had declined back to baseline values, similar to the results of Patient #2.

## Discussion

Clinical trials previously used hESC-derived RPE cells in an effort to treat atrophic AMD and Stargardt's disease^19–21^, while RPE cells derived from iPSCs were used as a treatment for wet-AMD^22^. Our study confirmed that subretinal injections of hESC-derived RPE cells were safe and well tolerated as a treatment for wet-AMD. Morphological and functional examinations, in agreement with Schwartz and Mandai^19–22^, failed to reveal any significant safety concerns, such as tumor formation. Patients received immunosuppressants for 4 months, and signs of immunologic rejection, cystoid macular edema, or retinal neovascularization were not observed, which is consistent with reports by Schwartz et al.^19,20^.

There are two main approaches to deliver cells into the subretinal space. One is the injection of an RPE cell suspension and the other involves engrafting a monolayer of RPE cells seeded on a supporting membrane. Although some researchers reported that cell suspensions did not integrate well into the damaged areas^28^, it is widely accepted that cell suspension also resulted in marked improvement in visual function. More importantly, this approach is relatively easy and has been accomplished in current clinical trials. A group led by Dr. Massayo Takahashi (RIKEN, Wako-shi, Saitama, Japan) performed a comparison study. P21 RCS rats received cell transplantation either by human iPSC-RPE cell sheet transplants or by injections of human iPSC-RPE cell suspension into the subretinal space. No significant difference in the improvement in visual function was reported. Both groups showed significant improvement in ERG responses and preservation of outer nuclear layer when compared with contralateral eyes^29^. Thus it seems there is still some controversy concerning about the two deliver methods. Considering the ease of application, the cell suspension injection also seems appropriate in clinical trial.

Wet-AMD patients present specific problems concerning the ideal delivery of transplant cells. The CNV membrane should, as far as possible, be removed and presents an additional challenge compared to the methods used by Schwartz et al.^19,20^ to treat dry-AMD. We performed a retinotomy of the peripheral retina to remove the CNV membrane in contrast to the methodology of Mandai et al.^22^, wherein a retinotomy incision was made around the vascular arch and near the diseased area. We injected a cell suspension into the subretinal space instead of inserting a cell sheet, and care was taken to select a retinotomy site that was sufficiently far enough away from the macular area (the transplant target) so that cell diffusion into the vitreous cavity was kept to a minimum. We performed subretinal injections using a 39 G needle after a silicone oil tamponade and, because the cell suspension is heavier than the silicone oil, the patient was required to remain face-up for 12 h after the surgery. This allowed the subretinal fluid to be effectively absorbed, thus avoiding leakage of injected cells and further minimizing cell diffusion into the vitreous. The face-down position was assumed after this 12 h period, a general requirement after vitrectomy for giant retinal tears^30^. Our clinical examination showed that transplanted cells were not visible in the vitreous cavity or anterior chamber, demonstrating that our modified surgical strategy successfully minimized cell leakage from the atrophic area. Imaging with SD-OCT in two patients showed that the ESC-RPE cells tended to form a sheet in the subretinal space over time. Although dark brown to black subretinal pigmentation was not present within the new RPE-like layer in fundus photography, the donor cells did form a highly reflective line in the SD-OCT image and this was similar to the host RPE layer. Furthermore, the neural retina outside the region of the transplanted RPE-like layer was much thinner compared to regions in the area of the transplanted RPE-like layer. This result, which parallels that found in the present animal study and those of others^31^, suggests that the injected cells may play a supportive/nutritional role for the neural retina.

We used the ETDRS visual acuity method and Snellen charts to test for any change or improvement in visual function. Visual acuity in the treated eyes using ETDRS had improved by 16 letters (13, 11, 26 respectively for Patient #1, 2, 3) at 12 months post-transplant, and it improved from just HMs preoperatively to 20/400 in two patients and remained stable in one patient based on the Snellen system. It should be noted that because our patients had a large central scotoma and poor visual function preoperatively, this hindered our ability to perform reliable measurements. We used mfERG to identify lesions localized to the macula and FVEP to provide information regarding the functional integrity of the entire visual system at the cortical level^26,32^. Both methods indicated that a functional decline did not occur during the 12-month follow-up period. Additionally, the central response density of the mfERG had increased in all three patients by 12 months post-transplant. The P2 amplitudes of the FVEP increased in one patient and remained stable in the other two patients. These results suggest that our strategy is safe and resulted in some limited functional improvement for wet-AMD patients over the 12-month period. Due to the small sample size enrolled in the study and the lack of a control group with only CNV removal, it is difficult to conclude that hESC-RPE cells implantation on its own leads to the visual improvement seen in our phase I clinical study. A randomized clinical trial of Submacular Surgery Trials (SST) that evaluated surgical removal versus observation of the subfoveal CNV secondary to wet-AMD^9^ showed that the median visual acuity loss from baseline over 12 months was 2.0 lines (10 letters) in the observation group (*n* = 210) and 1.8 lines (8 letters) in the surgery group (*n* = 214) . This study suggests that submacular surgery to remove CNV did not improve or preserve visual acuity and is not recommended for patients with wet-AMD. Additionally, this study also indirectly supports our assertion that improved vision in our trial is not solely due to CNV removal. Schwartz et al.^20^ reported a median safety period of 22 months following a hESC-RPE cell suspension transplanted into patients with dry-AMD and Stargardt's disease. BCVA improved in 10 eyes (56%), improved or remained the same in seven eyes (39%), and decreased by more than ten letters in one eye (5%). These results have provided strong evidence of the medium- to long-term safety, graft survival, and possible biological activity of a hESC-RPE cell suspension injection into individuals with AMD. Our animal study and clinical trial showed that implanted hESC-RPE cells form a thin layer like distribution and improved visual outcomes. Whether this rescue of vision is due to replacing the lost RPE cells or the paracrine effects of growth factors secreted by the implanted hESC-RPE cells remains to be determined. It is certainly possible that a combination of both mechanisms are responsible for the rescue of photoreceptors.

We did not observe any adverse complications, in particular, macular edema, which occurred in the study of Mandai et al.^22^ Their study, on the basis of BCVA results, concluded that vision had neither improved nor worsened after iPSCs-derived RPE cell sheet transplants, in contrast to our results demonstrating some improvement. The lack of persistent edema in our patients may account for the differences in visual function seen between these two studies. The iPSC-RPE sheets, which are grown on gelatin and are enzymatically dissolved before implantation, provided an RPE monolayer scaffold without a potential immunogenic or functional disturbance^29^. These comparison studies showed no significant differences when using an iPSC-RPE sheet or suspension transplants. In our study, suspended cells form a thin layer in the subretinal space in RCS rats and in wet-AMD patients, consistent with previous reports in dry-AMD patients^19,20^. Intraocular surgery by itself, including vitrectomy, retinectomy, and implantation, has been associated with intraocular inflammation as indicated by clinical findings, such as post-operative cystoid macular edema, epiretinal membrane formation, and vitreous or aqueous cell and proliferative vitreoretinopathy. Cell suspension injections with minimal surgical trauma, minimizes the amount of surgical manipulation during the implant procedure and, therefore, is less likely to result in edema.

Our study provides some promising early results concerning the use of hESC-RPE cells to alleviate wet-AMD. However, it is clear that further follow-up tests are necessary to determine the long-term viability of the transplanted cells and any further changes in visual function. Expanding the study to include more patients and, importantly, to include patients at the early stages of AMD are needed to verify the long-term benefits of cell transplant methodology.

## Materials and methods

### Q-CTS-hESC-2 cell culture and differentiation into RPE in a xeno-free system

The Q-CTS-hESC-2 cell line was provided by the State Key Laboratory of Stem Cells and the Reproductive Biology, Institute of Zoology, Chinese Academy of Sciences, and was tested and authenticated by the Chinese National Institutes for Food and Drug Control (Report Number SH201402035)^24^. The Q-CTS-hESC-2 cell line was maintained in xeno-free Essential 8™ Medium (A1517001, Gibco) without feeders. The protocol for the differentiation of RPE cells has been described previously^25^. Briefly, day 0 was set as the day on which the Q-CTS-hESC-2 cells were cultured in basic hESC medium without bFGF. Q-CTS-hESC-2 colonies were allowed to become super-confluent and had formed pigment foci by ~ day 20–30. When the pigment foci reached at least 1 mm in diameter they were mechanically excised and placed in 6-well plates coated with recombinant human vitronectin-N (rhVTN-N) (A14700, Gibco), and were described as hESC-RPE cells (Q-CTS-hESC-2-RPE cells) at passage 0 (Supplementary Fig. S1). After three passages (~125 days), hESC-RPE cells were identified for clinical use (see supplementary methods). The culture medium for the hESC-RPE cells consisted of Knock Out DMEM CTS (KO-DMEM, A1286101, Invitrogen), 20% Knock Out SR xeno-free CTS Knockout serum replacement (KSR, 12618012, Invitrogen), 1% non-essential amino acid solution, MEM NEAA (11140-050, Gibco), 1 mM CTS GlutaMAX-1 supplement L-Glutamine (A12860-01, Invitrogen), and 0.1 mM β-mercaptoethanol (Sigma21985023, Gibco).

### Subjects

We enrolled three patients diagnosed with wet-AMD on the basis of their clinical history, eye examinations, SD-OCT, fundus fluorescein angiography (FA) and ICGA. Patients met the following inclusion criteria: (i) age ranging from 50 to 75 years old and visual acuities ranging from HMs to 35 letters with ETDRS; (ii) typical subfoveal lesions, including CNV, fibrotic scars, or an RPE tear; and (iii) ineffective conventional therapies, including anti-VEGF administrations. Exclusion criteria included; evidence of other eye diseases, such as an advanced cataract that could compromise the interpretation of visual acuity; an inability to return for follow-up examinations according to a pre-planned schedule; a history of intraocular surgery.

### hESC-RPE cell safety control

hESC-RPE cell culture was performed using Good Manufacturing Practices in the Cell Biology Therapy Center, Southwest Hospital, Third Military Medical University. This center was awarded Good Manufacturing Practices certification and was qualified for the production of hESC-RPE cells. A biological safety study was conducted according to our previous methods^24^, an included karyotype analysis, tests for Hepatitis A, B, and C, Human Immunodeficiency Virus, Treponema pallidum, bacteria, fungi, mycoplasma, and endotoxins.

### Surgical procedure

The procedure for the surgical removal of submacular CNV closely followed that described by the SST research group study^33^. We carried out a 3-port pars plana vitrectomy followed by the creation of a complete posterior vitreous detachment and total vitreous excision. A 41-gauge injection cannula (Bausch & Lomb Storz, USA) was then used to infuse a balanced salt solution into the subretinal space in order to form a retinal detachment in the superior-temporal retina. Peripheral retinotomy was performed in the temporal quadrant and the CNV membrane carefully removed. The vitreous body was filled with perfluorodecalin to reattach the retina and photocoagulation around the retinal tear used to tack the retina in place. This was followed by a silicon oil-perfluorodecalin exchange to tamponade the vitreous body. Using a 39-gauge cannula and continuous visualization, the Q-CTS-hESC-2-RPE cell suspension was slowly injected into the subfoveal space, thereby creating a localized dome-shaped retinal detachment in the macular area (Supplementary Movie S1). The total volume of the injected cell suspension did not exceed 100 μl and contained a total of 10^6^ cells. After the surgery, the patient remained in a supine position overnight until the subretinal fluid had been absorbed, then the patient assumed a face down position and maintained this for 1 week.

### Immunosuppression protocol

Administration of immunosuppressive drugs followed the studies of Schwartz et al. *(19*,*20)*, albeit with minor modifications. One week before surgery, tacrolimus (serum concentration 10 ng/ml), mycophenolate mofetil (200 mg, twice/day), and prednisone (30 mg, once/day) were administered and continued for 1 month. One month post-transplant, tacrolimus was slowly reduced to a serum level of 3–7 ng/ml and by 2 months post-transplant, prednisone was decreased to 15 mg/day and mycophenolate mofetil was discontinued. Prednisone was discontinued at 3 months post-transplant and tacrolimus was reduced to a serum level of 3 ng/ml. All immunosuppression drugs were discontinued at 4 months post-transplant.

## Electronic supplementary material

Supplementary Infomation

Supplementary Video S1

## Electronic supplementary material

**Supplementary Information** accompanies the paper at (10.1038/s41421-018-0053-y).
