# Supplementary material for: Human embryonic stem cell-derived retinal pigment epithelium transplants as a potential treatment for wet age-related macular degeneration
Source: Cell Discov. 2018 Sep 11;4:50. doi: 10.1038/s41421-018-0053-y (PMC6143607; doi:10.1038/s41421-018-0053-y)
Supplement: Supplementary file 1 — Supplementary Infomation [file 41421_2018_53_MOESM1_ESM.pdf]

Supplementary Information

Supplementary Methods

Whole exome sequencing

Q-CTS-hESC-2-RPE samples were performed whole exome sequencing in targeted exon regions by the Beijing Genomics Institute (BGI). The variation sites for oncogenes and tumor suppressor genes were furtherly performed sanger sequencing to detect the possible alterations on tumorigenicity according to the whole exome sequencing results.

Teratoma formation

100 μl of a hESC or hESC- RPE cell suspension (1×10^7 cells) was subcutaneously implanted into the groin of SCID mice (regardless of sex). Mice were maintained under pathogen-free conditions at the animal facility of the Third Military Medical University and received humane care according to the criteria outlined by the requirements of the Laboratory Animal Welfare and Ethics Committee of the Third Military Medical University. We monitored the tumor size and condition of the mice weekly. The animals were anesthetized with overdose three months later and the tissue was examined by a pathologist to identify microscopic pathological changes and evidence of tumor formation.

Flow cytometry

Flow cytometry was used to identify the purity of hESC-RPE cells according to our previous methods and other reports [1-3]. Briefly, cells cultured in growth medium or differentiation medium were detached using HyQTase (Thermo Fisher Scientific) and collected. Cells were blocked with CD32/16 (Bio- Legend) and then incubated for 30 min at 4°C with primary antibodies to surface markers (1:30) or an isotype control (1:30; BioLegend). Cells were rinsed with staining buffer (eBioscience) and a permeabilization buffer (eBioscience) after each step in the procedure. Cells were counted using a FACS Calibur Flow Cytometer and at least 10,000 events were collected for each sample; samples were analyzed using FlowJo software (FlowJo, Ashland, OR, USA).

Transplantation of RPE cells into RCS rats

Thirty-five post-natal day 21 RCS rats (male and female) were maintained on a 12 h light/dark cycle. Animals were anesthetized using a mixture of 10 mg/kg ketamine (Sigma-Aldrich, St. Louis, MO, USA) and 1 mg/kg xylazine (Sigma-Aldrich), then a small scleral incision made and a Q-CTS-hESC-2-RPE cell suspension containing ~ 1 $\times 10^5$ cells in 2 μl of saline injected via a fine glass pipette (Hamilton) into the subretinal space (n=18). The cornea was punctured to reduce intraocular pressure and to reduce the efflux of cells. 0.01 M phosphate-buffered saline (PBS) injections were used as a vehicle control (n= 17). The right eyes served as the treatment eyes, whereas the left eyes were untreated. The rats were immunosuppressed by the addition of 210

mg/L cyclosporine A (Sandoz, Camberley) to the drinking water 24 h prior to the cell transplant and for the remainder of the experiment.

Electroretinograms (ERG)

Full-field ERGs were recorded after overnight dark adaptation as outlined previously [4]. Briefly, rats were anesthetized and the pupils dilated with Compound Tropicamide Eye Drops. Seventy eyes were tested, and consisted of three groups, a Q-CTS-hESC-2-RPE cell transplant group, saline injection (sham) group, and an untreated group. The rats were test with sequences of scotopic flashes (−20 db and 0 db), and the means for the A- and B-wave responses (maxima measurements for five flashes) were analyzed for each sequence.

Reverse transcription-quantitative polymerase chain reaction (RT-qPCR)

Total RNA was extracted using an RNAprep Pure Cell Kit (Sangon Biotech) according to the manufacturer's instructions. Total RNA (~ 1–2 μg per 20 μl reaction) was reverse transcribed using a PrimeScript® RT Reagent Kit (Takara). Quantitative PCR was performed by a CFX96 Real-Time PCR System (Bio-Rad) using a SYBR Green qPCR Mix (Dongsheng Biotech) according to the manufacturer's instructions. Relative expression levels were normalized to GAPDH and were calculated using the 2−ΔΔC(t) method. All primers are listed in supplementary Table 1. RT-qPCR assays for hESC markers: Nanog, OCT4 and SOX2 and hRPE markers: RPE-65, PAX-6, MITF, bestrophin and CRALBP were normalized to the level of expression observed in the 100% hESC sample, which served as the zero set point.

Immunostaining

Cells grown on cover-slips were fixed with 4% paraformaldehyde for 15 min, permeabilized using 0.1% Triton X-100 in PBS for 15 min, and blocked for 60 min in 5% goat serum. Primary antibodies against Pax6, MITF, ZO1, and RPE cell markers including Cralbp, Bestrophin-1 and RPE65 were diluted in the same blocking buffer and incubated with the samples overnight at 4°C, washed, and then incubated with a fluorescently coupled secondary antibody for 1 h at room temperature. The nuclei were stained with 4,6-diamidino-2-phenylindole (DAPI; Invitrogen). Fluorescent images were acquired with a confocal microscope (Zeiss LSM 700, Carl Zeiss; software: Zen lite 2012).

Mouse anti-human mitochondria (1:200, Abcam) was used as primary antibodies to detect the transplanted hESC-RPE cells, rabbit anti-human or anti-rat RPE65 (1:100, Abcam) marked host or donor RPE cells and ki67 (1:250, Chemicon) labeled proliferative cells, then sections were incubated in three secondary antibodies. To compare the degree of outer nuclear layer (ONL) preservation between hESC-RPE and vehicle groups, sample areas were chosen from the center of the transplanted area and a region opposite this in order to quantify ONL thickness (n= 5, five rats were sacrificed from one group at each time point, three random sections that containing the typical transplant areas were selected from one rat).

Fundus Fluorescein Angiography (FFA), Indocyanine Green Angiography (ICGA),

and Synchronized Angiography

A solution containing 3 ml of 10% sodium fluorescein (Alcon Laboratories, Inc., USA) and 2 ml of 25 mg indocyanine green (Dandong Yichuang Pharmaceutical CO.,LTD, China) was injected intravenously (within 5 s) into the chephalic vein of the forearm, followed by a flush of normal saline. A confocal laser scanning system (Heidelberg retina angiography II, HRII, Heidelberg, Germany) was used to capture FFA and ICGA real-time fundus images at early, mid, and late time periods (1 – 30 min) after the intravenous injection. These procedures, imaging systems, and contrast dyes, are approved by the Food and Drug Administration.

Spectral Domain Optical Coherence Tomography (SD-OCT)

A Heidelberg Spectralis HRA+OCT device (Heidelberg Engineering, Germany) was used and all SD-OCT scans were performed by the same experienced operator. The retina was viewed through a dilated pupil and an internal fixation light used to center the scans on the fovea. Images were generated using a high-speed volumetric raster scan pattern over a 30°×25° area. Each raster scan consisted of 61 horizontal line scans spaced 127 μm apart. Each horizontal line scan was created from 1536 A-scans. The final horizontal line used in the analyses consisted of 9 B-scans that were averaged using the automatic real-time mode to reduce speckle noise. Two scanning sessions were performed by the same operator, with patients repositioned between sessions. An additional radial scan of the macular zone was performed that included 12 radial lines centered on the fovea. The SD-OCT scan at each visit included scans of the macular and subretinal transplantation area. All images were checked by an

experienced retinal specialist. Any subretinal reflection that was recognized as a transplanted cell group was marked and positioned according to the horizontal and radial lines, then the distribution of transplanted cells reconstructed according to the serial positioning marks.

FVEP and mfERG Test

Multifocal electroretinography (mfERG) elicited by a Veris system (Electro-Diagnostic Imaging, Inc., Burlingame, CA, U.S.A.) was used to test the local function of the macula and FVEPs elicited by an Espion system (Diagnosys LLC, Lowell, MA, U.S.A) were used to functionally test the visual pathway. All tests were performed according to the International Society for Clinical Electrophysiology of Vision (ISCVE) Standards [5,6] and repeated three times in every test. The test protocols we used for each kind of investigation were as follows;

mfERG: The "M-sequence" algorithm was used to control the temporal sequence of change between the light and dark stages of each stimulus hexagon. The stimulus field contained 103 hexagons with a field diameter of 40°. The pupils were fully dilated, bipolar corneal contact electrodes attached, and a real-time fundus camera used to monitor eye movements during the test. Monocular stimulation was used for a four min recording session band-pass filtered at 10-300 Hz. The standard measurement for mfERG amplitude density is the trough-to-peak amplitude (nv) of the N1 and P1 responses over its hexagon in degrees (nv/deg^2). Groups of responses from the central to the peripheral retina can be divided into rings 1 to 6.

FVEP: a flash VEP was elicited by a brief flash (< 5 ms) in an integrating bowl (ganzfeld) that subtended a visual field of 20° (scotopic conditions). The strength (time-integrated luminance) of the flash stimulus was 3 cd.s.m^{-2}, duration 5 ms, at a flash rate of 1.0 Hz and a screen refresh rate of 17 ms. A single recording channel with a midline occipital active electrode (Oz) and reference electrode at Fz were used according to the International 10/20 system; the pupils were not dilated. Recordings were band-pass filtered from 1 to 100 Hz at a sampling rate of 1000 Hz. Flash amplitude measurements were taken from the N2 negative (90 ms) and P2 positive peaks (120 ms).

References

1 Osakada, F. *et al.* Toward the generation of rod and cone photoreceptors from mouse, monkey and human embryonic stem cells. *Nature biotechnology* **26**, 215-224, doi:10.1038/nbt1384 (2008).

2 Wu Wei , Z. Y., Li Zhengya, Li Qiyou, Xu Haiwei , Yin Zheng Qin. Features specific to retinal pigment epithelium cells derived from three-dimensional human embryonic stem cell cultures —a new donor for cell therapy. *Oncotarget* **7**, 22819-22833 (2016).

3 Schwartz, S. D. *et al.* Embryonic stem cell trials for macular degeneration: a preliminary report. *Lancet* **379**, 713-720, doi:10.1016/s0140-6736(12)60028-2 (2012).

4 Huo SJ, L. Y., Xie J, Li Y, Raisman G, Zeng YX, He JR, Weng CH, Yin ZQ. Transplanted olfactory ensheathing cells reduce retinal degeneration in Royal College of Surgeons rats. *Curr Eye Res.;*. **37** (2012).

5 Odom JV, B. M., Brigell M, Holder GE, McCulloch DL, et al. ISCEV standard for clinical visual evoked potentials: (2016 update). *Doc Ophthalmol* **133**, 1-9. (2016).

6 Hood DC, B. M., Brigell M, Keating D, Kondo M, et al. . ISCEV standard for clinical multifocal electroretinography (mfERG) (2011 edition). . *Doc Ophthalmol* **124**, 1-13. (2012).

Supplementary Figures

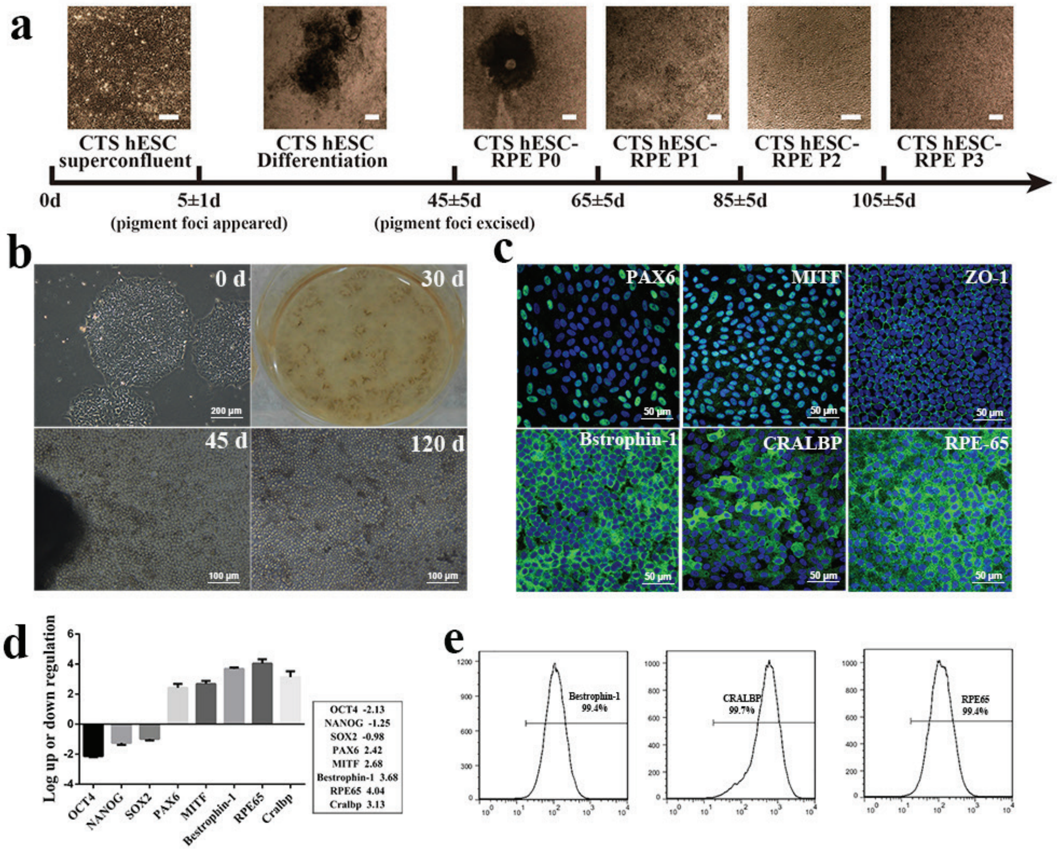

Supplementary Fig. S1. Figures show the differentiation of clinical-grade human embryonic stem cells (Q-CTS-hESC-2) into retinal pigment epithelium (hESC-RPE) in a Xeno-free culture system. (**a**) The sequence of Q-CTS-hESC-2 differentiating into RPE cells (scale bar = 200 μm). (**b**) Morphologic characteristics of the induced cells at different times. On day 0, the hESCs had a normal shape within the colonies, and super-confluent pigmented cells were obtained by culturing in a six-well plate for 20~30 days. Pigmented cell foci expanded and exhibited an epithelial phenotype at 45 days. After three passages, the hESC-RPE cells showed a confluent cobblestone monolayer when viewed with Hoffman modulation contrast microscopy at 120 days; these cells were used for the later studies. (**c**) Cells were positive for Pax6, MITF,

ZO-1, CRALBP, Bestrophin-1 and RPE65 (green; scale bar = 50 μm). Nuclei were stained with DAPI. (**d**) The expression of hESC-related genes, OCT4, NANOG, SOX2, markedly decreased while RPE cell-related genes, PAX6, MITF, Bestrophin-1, RPE65 and CRALBP, were highly expressed (n = 3). (**e**) The purity of the hESC-RPE cell cultures was more than 99% when tested by FACS after staining the cells with the RPE markers, bestrophin-1, CRALBP, and RPE65.

DAPI = 4,6-diamidino-2-phenylindole; FACS = Fluorescence Activated Cell Sorting

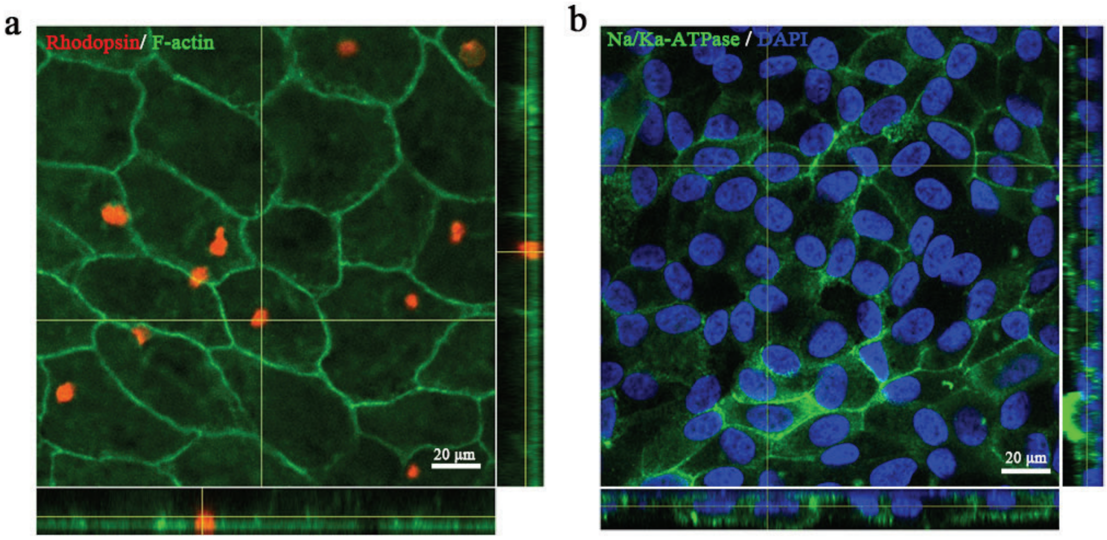

Supplementary Fig. S2. The functions of POS phagocytosis and polarity testing in hESC-RPE cells.

(a) Z-stack fluorescent images with cross-section side views showed the location of internalized POS in hESC-RPE cells at day 120. (b) Na/ K-ATPase immunostaining showed Na, K-ATPase was expressed at the apical membrane in hESC-RPE cells.

POS = photoreceptor outer segment

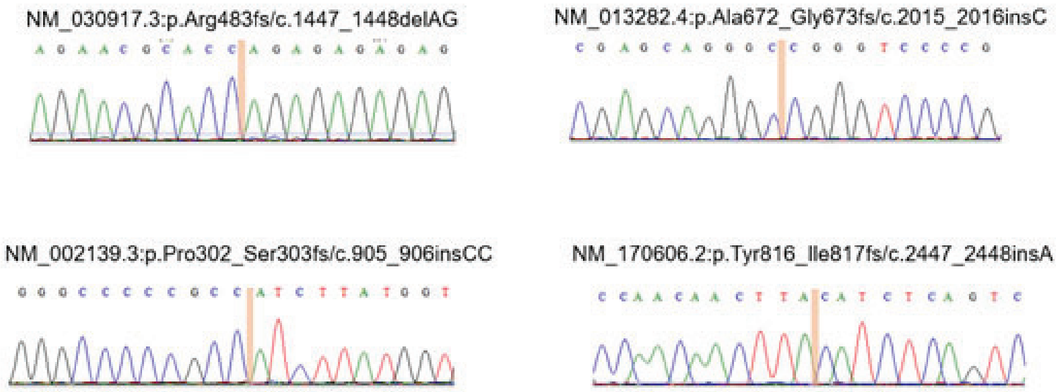

Supplementary Fig. S3 The Sanger sequencing vertification of the Whole Exome Sequencing. Four related mutation sites were identified based on the results of the sequencing. Then these 4 mutation sites were verified with Sanger PCR method, no mutations in these candidate sites for oncogenes or tumor suppressor genes were found in these Q-CTS-hESC-2-RPE cells.

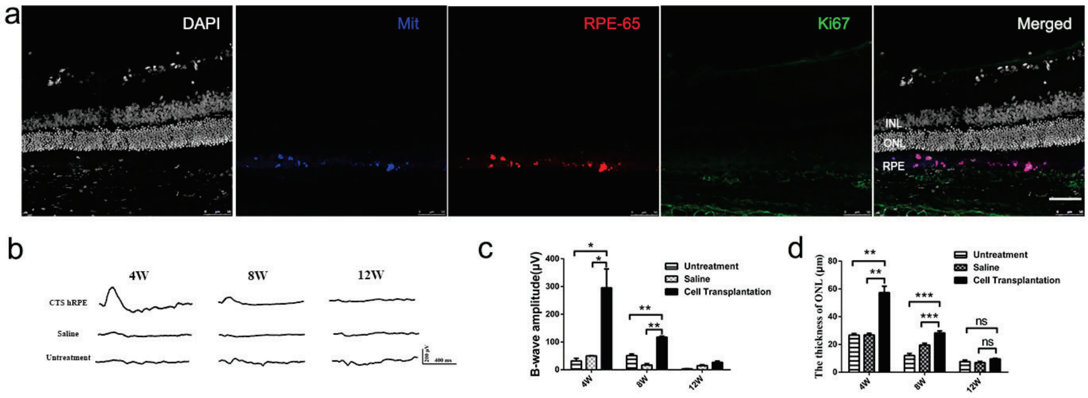

Supplementary Fig S4. Q-CTS-hESC-2-RPE cell transplants in Royal College of Surgeons (RCS) rats. (**a**) hESC-RPE cells (stained with a human-specific mitochondria antibody, Mit) were still present in the subretinal space of RCS rats 4 weeks after transplantation, and cell also expressed RPE65 but were not stained with Ki67, indicating RPE phenotype without proliferation. Note the INL, ONL and RPE layering in the merged figure. (**b**) Flash electroretinography (FERG) recordings from

RCS rats after CTS hRPE transplants were compared to saline injections and untreated animals. (**c**) Graph shows the analysis of the b wave amplitude recorded in the FERG at 4, 8 and 12 weeks after cell transplantation (* $p < 0.001$, ** $p < 0.01$). (**d**) The thickness of the outer nuclear layer (ONL) in the hESC-RPE transplant group was markedly thicker at 4 and 8 weeks compared with the saline or untreated groups. (** $p < 0.01$, *** $p < 0.05$, $n = 15$).

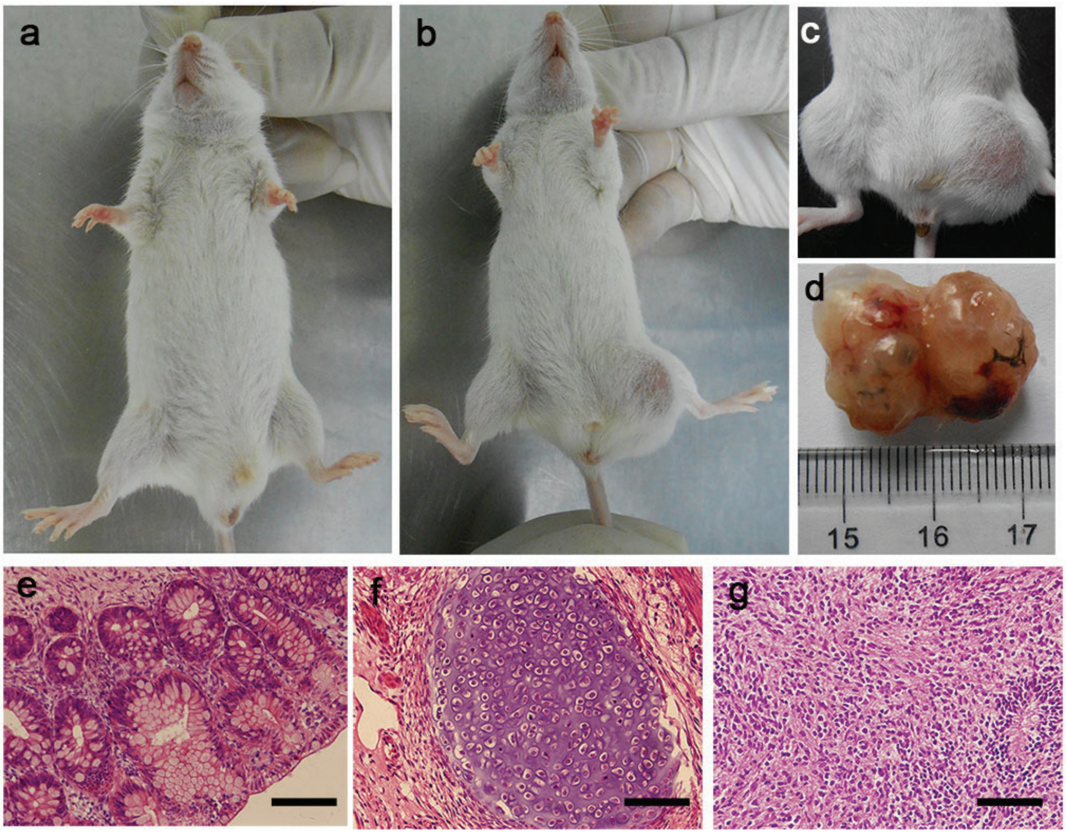

Supplementary Fig. S5. Teratoma formation by Q-CTS-hESC-2-RPE and hESC cells in SCID mice. (**a**) No teratomas were formed up to three months after the injection of Q-CTS-hESC-2-RPEcells ($n = 46$). (**b & c**) Teratomas were formed in the groin in 65% of the SCID mice ($n = 13/20$) that received a subcutaneous injection of hESCs. (**d**) Gross anatomical appearance of a hESC formed teratoma. (**e-g**) Presence

of the endodermal, mesodermal, and ectodermal layer derivatives were observed in the hESC formed teratoma, and included intestine (**e**), bone (**f**) and neuron (**g**) tissue. Bar = 50 μm

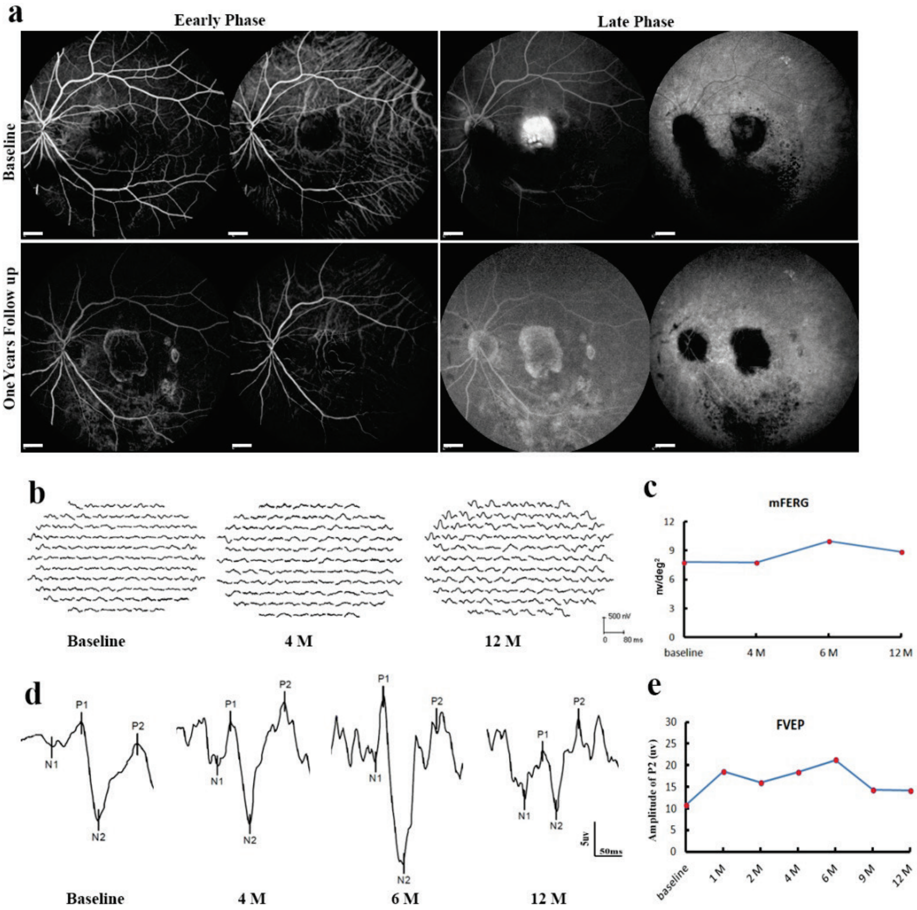

Supplementary Fig. S6. Angiographic characteristics and physiological changes in Patient #2 after CNV removal and hESC-RPE transplantation. (**a**) FA (left side of pair) and ICGA images (right side of pair) show the retinal and choroidal vascular changes. The foveal fibrotic CNV blocked fluorescence in early phase and fluorescence leakage into the corresponding tissue can be seen in late phase before surgery. One year later, a CNV was present after FA and ICGA examination in early phase.

Additionally, fluorescence leakage was also not observed in late phase (scale bar = 1mm). (**b**) The mfERG waves before (baseline) and after (4 and 12 months follow-up) transplantation. (**c**) Analysis of amplitude density in the central hexagon (ring 1) representing foveal visual function. (**d**) The FVEP waveform at different time points after transplantation. (**e**) Graph shows the changes in the amplitude of the P2 wave induced by FVEP.

FA = fundus fluorescein, ICGA = indocyanine green fluorescein angiography, mfERG = multifocal electroretinography, FVEP = flash visual evoked potentials

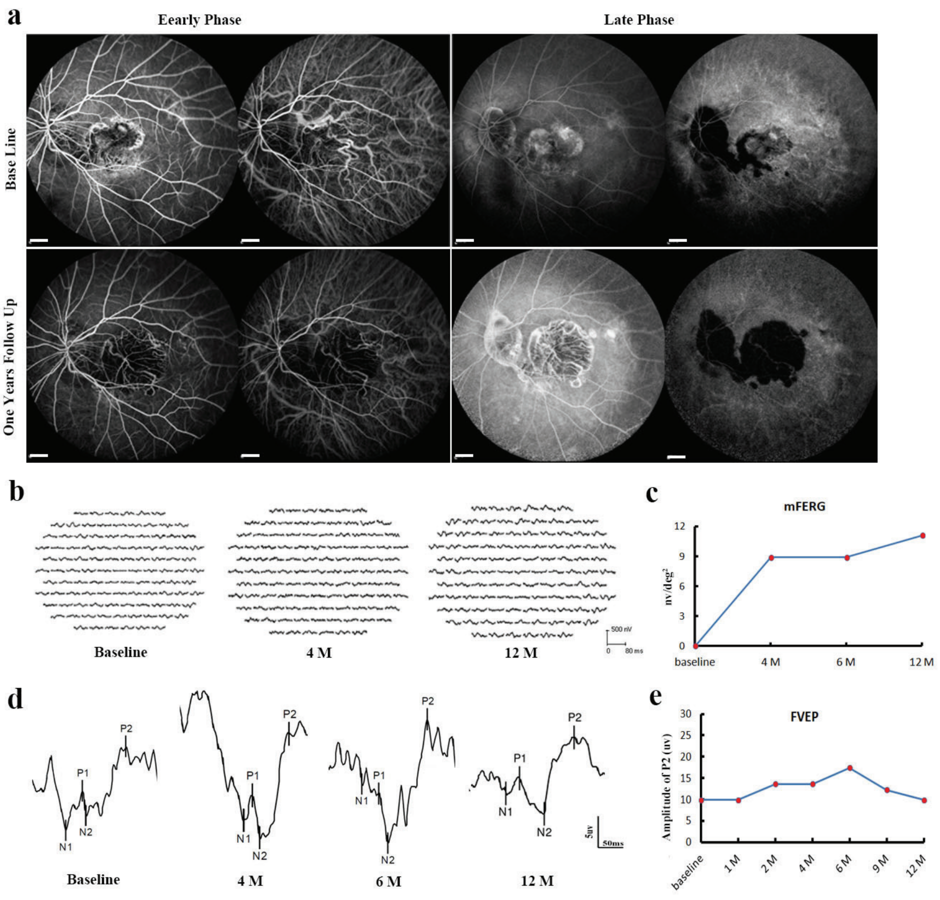

Supplementary Fig. S7. Angiographic characteristics and physiological changes in Patient #3 after CNV removal and hESC-RPE transplantation. (**a**) The foveal fibrotic

CNV blocked fluorescence in early phase and fluorescence leaked into the corresponding tissue in late phase before surgery. One year later, a CNV was not observed with FA and ICGA examination in early phase, and fluorescence leakage was not found in late phase (scale bar = 1mm). (**b**) The mfERG waves before (baseline) and after (4 and 12 months follow-up) transplantation. (**c**) Changes of amplitude density in the central hexagon (ring 1). (**d**) The FVEP waveform at different time points after transplantation. (**e**) Changes in the amplitude of the P2 wave induced by FVEP.

Supplementary Movie S1. Video of CNV removal and hESC-RPE cell injection.

Supplementary Table S1. Q-CTS-hESC-2-RPE Safety Testing

Supplemental Table 1 Q-CTS-hESC-2-RPE Safety Testing

Test	Specification	Clinical hRPE Cell
Fungus	Negative	Negative
Bacterium	Negative	Negative
Mycoplasma	Negative	Negative
Hepatitis B Virus (HBV)	Negative	Negative
Human Immunodeficiency Virus (HIV)	Negative	Negative
Hepatitis C Virus (HCV)	Negative	Negative
TreponemaPallidum (TP)	Negative	Negative
Endotoxin	< 0.50 EU/mL	＜0.5 EU/ml
